# Particularidades Clínicas e Ecocardiográficas da Cardiomiopatia Hipertrófica em uma População Brasileira e seu Impacto Prognóstico

**DOI:** 10.36660/abc.20240640

**Published:** 2025-04-17

**Authors:** Georgina Del Cisne Jadán Luzuriaga, Edmundo Arteaga-Fernandez, Viviane Tiemi Hotta, Barbara Ianni, Luciano Nastari, Felix Ramires, Guilherme Wesley Peixoto da Fonseca, Charles Mady, Fábio Fernandes, Juliano Novaes Cardoso

**Affiliations:** 1 Instituto do Coração Hospital das Clínicas Universidade de São Paulo São Paulo SP Brasil Instituto do Coração do Hospital das Clínicas da Faculdade de Medicina da Universidade de São Paulo, São Paulo, SP – Brasil; 2 Escola de Educação Física e Desporto Universidade de São Paulo São Paulo SP Brasil Escola de Educação Física e Desporto da Universidade de São Paulo, São Paulo, SP – Brasil; 3 Universidade de São Paulo Faculdade de Medicina São Paulo SP Brasil Universidade de São Paulo Faculdade de Medicina – Unidade Clínica de Miocardiopatias e Doenças da Aorta, São Paulo, SP – Brasil; 4 Faculdade Santa Marcelina São Paulo SP Brasil Faculdade Santa Marcelina – Cardiologia, São Paulo, SP – Brasil; 5 Hospital Santa Marcelina São Paulo SP Brasil Hospital Santa Marcelina, São Paulo, SP – Brasil

**Keywords:** Cardiomiopatia Hipertrófica, Mortalidade, Ecocardiografia, Volume Sistólico

## Abstract

**Fundamento:**

A cardiomiopatia hipertrófica (CMH) apresenta alterações ecocardiográficas importantes para diagnóstico e prognóstico. Os dados da literatura nacional são escassos.

**Objetivo:**

Avaliar, em uma coorte brasileira de pacientes com CMH, as características clínicas, ecocardiográficas e a evolução da doença.

**Métodos:**

Coorte retrospectiva de pacientes com CMH e idade ≥ 18 anos. Foram excluídos pacientes com estenose aórtica moderada ou importante e aqueles submetidos à redução septal. O nível de significância adotado na análise estatística foi de 5%.

**Resultados:**

Foram incluídos 1244 pacientes, entre os anos de 2010 e 2022, com tempo médio de seguimento de 7,7 anos ± 4,5, sendo 53,6% homens. A idade média foi de 54,6 anos ± 16,5, e a fração de ejeção do ventrículo esquerdo (FEVE) média foi de 65,8% ± 7,6. A FEVE ≤ 50% foi observada em 5,8% dos pacientes, a forma assimétrica em 88,7% e a hipertrofia septal em 85,4%. Movimento sistólico anterior da valva mitral foi encontrado em 30,1% dos pacientes, obstrução da via de saída do ventrículo esquerdo em 30,7% e septo ≥ 28 mm em 7,2%. Apenas 1 paciente apresentou aneurisma ventricular. Fibrilação/flutter atrial ocorreu em 9,6% dos pacientes. A mortalidade geral ocorreu em 232 pacientes (1,3%/ano). Pacientes com peptídeo natriurético tipo B (BNP) > 200 pg/ml, átrio esquerdo ≥ 45 mm e FEVE ≤ 50% apresentaram maior mortalidade (p < 0,001). Além disso, idade e fibrilação/flutter atrial também foram relacionadas com mortalidade.

**Conclusões:**

A maioria dos pacientes apresentava FEVE > 50%, hipertrofia assimétrica e predomínio septal. BNP, diâmetro do AE, FEVE ≤ 50%, idade e fibrilação/flutter atrial foram associados com pior prognóstico.

## Introdução

A cardiomiopatia hipertrófica (CMH) é uma doença hereditária, caracterizada fenotipicamente por hipertrofia miocárdica que não pode ser explicada por condições sistêmicas, como hipertensão arterial sistêmica ou alterações metabólicas e sindrômicas.^1,2^ No adulto, o diagnóstico é definido pela espessura miocárdica diastólica ≥ 15 mm em qualquer localização do ventrículo esquerdo (VE), ou ≥ 13 mm em indivíduos com história familiar de parentes de primeiro grau com diagnóstico confirmado de CMH.^2-8^

O ecocardiograma transtorácico (ETT) é um exame acessível e fundamental para o diagnóstico inicial da CMH, a estratificação de risco e para o acompanhamento clínico.^8^ O método possibilita a avaliação morfológica e funcional cardíaca, além de identificar, quantificar e localizar a hipertrofia ventricular. Também permite a avaliação da presença de obstrução da via de saída do ventrículo esquerdo (OVSVE), de movimento sistólico anterior da valva mitral (SAM), de aneurisma apical ou de massas intracavitárias.^2,8^

A CMH apresenta duas formas clínicas: obstrutiva e não obstrutiva, de acordo com a presença de OVSVE, definida pela medida do gradiente ≥ 30 mmHg em repouso e ≥ 50 mmHg após manobras provocativas**,** como a manobra de Valsalva.^8^ Várias características avaliadas ao ETT estão relacionadas com o prognóstico, como as dimensões do átrio esquerdo (AE), a espessura miocárdica máxima septal, a fração de ejeção do ventrículo esquerdo (FEVE) e a presença de aneurisma apical do VE. A doença pode, em alguns casos, causar sintomas como dispneia, dor torácica, síncope e palpitações. Em pacientes considerados de alto risco para morte súbita, a CMH pode estar associada a uma maior mortalidade.^2,8,9-18^

O nosso estudo visa agregar conhecimento relacionado aos pacientes com CMH de nosso país, podendo entender melhor suas peculiaridades. Devido à importância do ETT na avaliação inicial, na estratificação de risco e no acompanhamento dos pacientes com CMH, o presente estudo teve como objetivo analisar as características clínicas, ecocardiográficas e a evolução em uma coorte brasileira de pacientes com CMH. Mesmo tratando-se de doença genética de padrão hereditário, é fundamental o conhecimento de particularidades relacionadas a variações demográficas, socioeconômicas e ambientais na expressão clínica da CMH.

## Material e métodos

O estudo avaliou uma coorte retrospectiva de pacientes com diagnóstico de CMH acompanhados em um hospital terciário da cidade de São Paulo, Brasil, no período de 2010 a 2022 a partir de dados de prontuário eletrônico. Pacientes menores de 18 anos, com estenose aórtica, submetidos a terapia de redução septal, cirurgia de válvulas cardíacas, transplante cardíaco ou com outras cardiomiopatias foram excluídos.

Foram analisados dados demográficos, clínicos e laboratoriais, além do ritmo no eletrocardiograma de repouso, ETT, Holter de 24 horas e a mortalidade por todas as causas.

### Ecocardiograma e medida da FEVE

As medidas das cavidades e dimensões cardíacas como diâmetro do AE, diâmetros e espessura miocárdica das paredes do VE foram realizadas pelo modo bidimensional no corte paraesternal longitudinal. Para as medidas do septo ventricular, foram excluídas estruturas do ventrículo direito como trabeculações, banda moderadora e crista supraventricular, além dos músculos papilares. A CMH do tipo assimétrico foi considerada quando a diferença entre um ou mais segmentos com hipertrofia miocárdica do VE foi > 2 mm avaliada ao ETT. A OVSVE foi definida na presença de gradiente na via de saída do VE ≥ 30 mmHg em repouso e ≥ 50 mmHg após manobra de Valsalva.^1,7^ Os exames foram realizados por médicos experientes em hospital de referência cardiológica em equipamentos de diferentes marcas ao longo de 12 anos de seguimento. Os exames foram realizados em aparelhos das marcas GE Healthcare Systems (Vivid I, Vivid E9, Vivid E95, iQ) e Phillips Healthcare (ie 33, Epic, CX e CVX).

### Análise estatística

Para processamento estatístico dos dados**,** foram utilizados os programas SPSS V26 (2019), Minitab 21.2 (2022) e Office Excel 2010. Utilizamos o teste T de Student não pareado para as variáveis contínuas e o teste qui-quadrado para as variáveis categóricas. O teste de Shapiro-Wilk foi utilizado para testar a normalidade das variáveis contínuas. As curvas de sobrevida foram analisadas com o método de Kaplan-Meier, e as diferenças na taxa de óbitos ao longo do tempo foram avaliadas usando o teste de log-rank. Modelos de regressão logística foram construídos para avaliar os fatores associados à mortalidade. Os valores de *odds ratio* (OR) e intervalo de confiança (IC) de 95% foram registrados. Associações significativas na análise univariada foram incluídas no modelo da análise multivariada. As variáveis contínuas foram apresentadas por média ± desvio padrão e as variáveis categóricas por frequência e porcentagem. O nível de significância adotado na análise estatística foi de 5%. O estudo foi aprovado pelo comitê de ética da instituição.

## Resultados

Foram incluídos 1244 pacientes no período de 2010 a 2022, com tempo médio de seguimento de 7,7 ± 4,5 anos. A idade média foi de 54,6 ± 16,5 anos, e 53,6% eram do sexo masculino. A fibrilação ou flutter atrial estavam presente em 9,6% dos pacientes. As características clínicas e laboratoriais estão descritas na Tabela 1.

**Tabela 1:** – Características clínicas e laboratoriais gerais da população total do estudo

	Todos n = 1244
**Idade, anos (DP)**	54,6 (16,5)
**Sexo**	
Masculino (%)	667 (53,6%)
Feminino (%)	577 (46,4%)
**Raça**	
Branca (%)	1.102 (88,6)
Preta (%)	67 (5,4)
Parda (%)	61 (4,9)
Amarela (%)	14 (1,1)
**Sinais vitais**	
FC (bpm)	64,9 ± 19,3
PAS (mmHg)	114,8 ± 33,7
PAD (mmHg)	71,5 ± 20,7
**Laboratório**	
BNP, pg/mL*	347,7 ± 492,7
Hemoglobina, g/dL	14,3 ± 1,7
Glicose, mg/dL	104,2 ± 29,3
LDL, mg/dL	116,2 ± 37,9
Ureia, mg/dL	36,9 ± 13,3
Creatinina, mg/L	1,0 ± 0,3
Potássio, mEq/L	4,4 ± 0,4
Sódio, mEq/L	139,9 ± 2,8
**ECG ^**†**^**	
Sinusal (%)	712 (85,6)
FA/flutter atrial (%)	80 (9,6)
Outros (%)	40 (4,8)

* 853 pacientes. ^†^ 832 pacientes. Os valores são expressos por média ± DP ou frequência e porcentagem. BNP: peptídeo natriurético tipo B; DP: desvio padrão; ECG: eletrocardiograma; FA: fibrilação atrial; FC: frequência cardíaca; PAD: pressão arterial diastólica; PAS: pressão arterial sistólica.

A avaliação do ETT revelou média do diâmetro do AE de 43,7 ± 7,3 mm e FEVE média de 65,8% ± 7,6, sem diferença estatística entre os sexos (p = 0,498). A FEVE > 50% foi observada em 1172 pacientes (94,2%), enquanto a FEVE igual ou menor que 50% foi observada em apenas 72 pacientes (5,8%). A forma assimétrica ocorreu em 1104 pacientes (88,7%), enquanto 1062 pacientes (85,4%) apresentavam hipertrofia de predomínio septal e 34 pacientes (2,7%) de predomínio apical. A espessura miocárdica do septo ventricular ≥ 28 mm foi evidenciada em 89 pacientes (7,2%), e em apenas 1 paciente foi identificado aneurisma ventricular apical. A presença de SAM foi encontrada em 374 pacientes (30,1%) e a OVSVE foi observada em 382 pacientes (30,7%).

**Figura 1:**
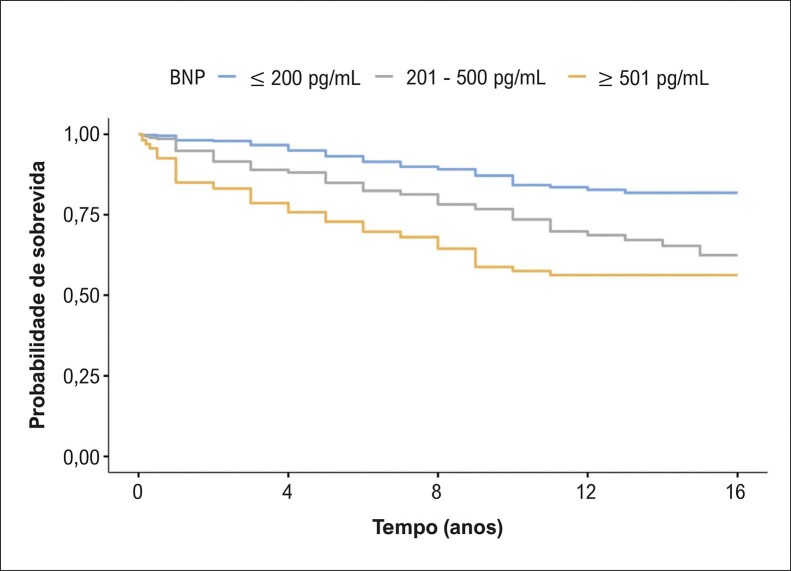
– Cardiomiopatia hipertrófica de acordo com os valores de BNP. Teste de log-rank, p < 0,001. BNP: peptídeo natriurético tipo B.

**Figura 2:**
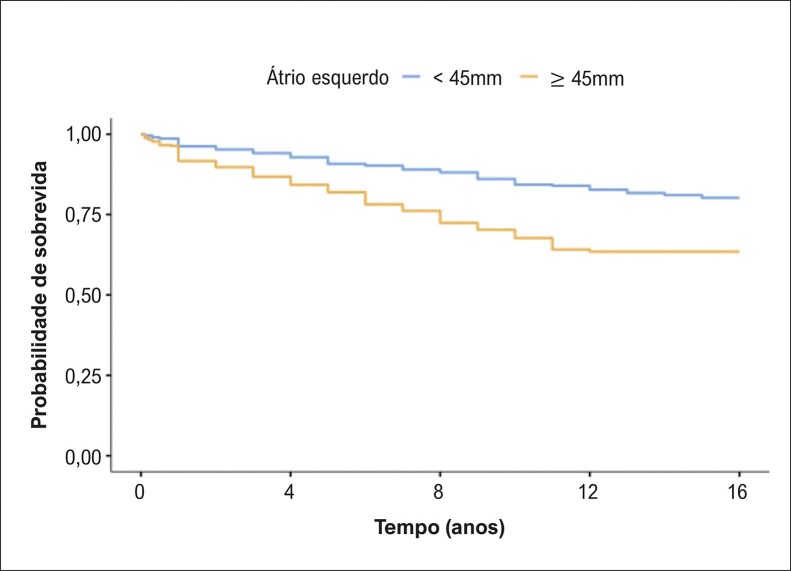
– Cardiomiopatia hipertrófica e átrio esquerdo ≥ 45 mm. Teste de log-rank p < 0,001.

Houve diferença entre os sexos na comparação da avaliação ecocardiográfica nos seguintes parâmetros: diâmetro do AE, septo interventricular, parede posterior e volume ventricular esquerdo (Tabela 2). Na avaliação quanto às faixas etárias, os pacientes com idade > 70 anos apresentaram com maior frequência BNP acima de 200 pg/mL, fibrilação ou flutter atrial e tamanho do AE ≥ 45 mm (Tabela 3). Durante o acompanhamento, com período médio de 7,7 anos (mínimo de 1 ano e máximo de 16 anos), a taxa de mortalidade foi de 18,6% (idade média de 62,0 ± 17,0 anos, sendo 53,9% do sexo feminino). A taxa de mortalidade anual foi de 1,3% entre os pacientes estudados. A curva de sobrevida revelou maior mortalidade para os pacientes com BNP > 200 pg/mL (Figura 1), diâmetro do AE ≥ 45mm (Figura 2) e FEVE ≤ 50%, com o teste de log-rank, p < 0,001. A análise univariada revelou que o sexo masculino foi associado com risco de óbito (OR 1,58, IC 95%: 1,18 a 2,10, p < 0,001). Também realizamos a análise multivariada, que evidenciou que as seguintes variáveis foram relacionadas à maior mortalidade: aumento da idade, aumento do diâmetro do AE, redução da FEVE e presença de fibrilação ou flutter atrial (Tabela 4).

**Tabela 2:** – Características ecocardiográficas da população total do estudo e por sexo

	Todos (n=1244)	Homens (n=667)	Mulheres (n=577)	p valor
**FEVE %**	65,8 ± 7,6	65,7 ± 7,7	66,1 ± 6,3	0,498
**AE (mm)**	43,7 ± 7,3	44,3 ± 7,9	43,0 ± 7,3	0,003
**SIV (mm)**	18,9 ± 5,7	19,3 ± 6,0	18,8 ± 5,6	0,002
**PPVE (mm)**	11,4 ± 2,7	11,6 ± 2,8	11,0 ± 2,8	0,012
**DDVE (mm)**	45,1 ± 6,0	46,3 ± 6,3	43,9 ± 5,5	<0,001
**DSVE (mm)**	28,7 ± 5,3	29,4 ± 5,7	27,8 ± 4,5	<0,001
**VDVE (ml)**	96,3 ± 40,0	103,3 ± 48,4	89,2 ± 26,4	<0,001
**VSVE, (ml)**	33,5 ± 17,8	35,8 ± 19,7	30,5 ± 12,4	<0,001
**IMVE (g/m^2^)**	161,6 ± 64,1	164,4 ± 68,8	160,9 ± 60,6	0,100
**Índice de espessura (mm)**	0,7 ± 0,2	0,7 ± 0,2	0,7 ± 0,2	0,109
**Segundo a simetria**				
Assimétrica (%)	1104 (88,7)	593 (88,9)	511 (88,6)	0,848
Simétrica (%)	140 (11,3)	74 (11,1)	66 (11,4)	
SAM (%)	374 (30,1)	198 (15,9)	176 (14,1)	0,754
**OVSVE**				
Presente	382	174	208	<0,001
Ausente	862	493	369	

Os valores são expressos por média ± desvio padrão ou frequência e porcentagem. AE: átrio esquerdo; DDVE: diâmetro diastólico do ventrículo esquerdo; DSVE: diâmetro sistólico do ventrículo esquerdo; FEVE: fração de ejeção do ventrículo esquerdo; IMVE: índice de massa do ventrículo esquerdo; OVSVE: obstrução da via de saída do ventrículo esquerdo; PPVE: parede posterior do ventrículo esquerdo; SAM: movimento anterior sistólico da valva mitral; SIV: septo interventricular; VDVE: volume diastólico do ventrículo esquerdo; VSVE: volume sistólico do ventrículo esquerdo.

**Tabela 3:** – Características por faixas etárias

	Todos	Idade 18 a 45 anos	Idade 46 a 70 anos	Idade ≥ 71 anos	p valor
**BNP**	n=853 (%)	n=247*(%)	n=464†(%)	n=143^‡^(%)	
≤ 200 pg/mL	422 (49,5)	122 (49,4)	253 (54,6)	47 (32,9)	<0,001
201 to 500 pg/mL	271 (31,8)	69 (27,9)	146 (31,4)	57 (39,9)	
≥ 501 pg/mL	160 (18,8)	56 (22,7)	65 (14,0)	39 (27,3)	
**ECG, ritmo**	n=832(%)	n=228 (%)	n=455 (%)	n=149 (%)	
Sinusal	713 (85,7)	218 (95,6)	378 (83,1)	117 (78,5)	<0,001
FA/flutter atrial	80 (9,6)	3 (1,3)	55 (12,1)	22 (14,8)	
Outros	39 (4,7)	7 (3,1)	22 (4,8)	10 (6,7)	
**Ecocardiograma**	n=1244(%)	n=372 (%)	n=658 (%)	n=214 (%)	
AE ≥ 45 mm	527 (42,4)	113 (30,4)	307 (46,7)	107 (50,0)	<0,001
SIV ≥ 28 mm	89 (7,2)	54 (14,5)	32 (4,9)	3 (1,4)	<0,001
FEVE < 50%	44 (3,5)	17 (4,6)	23 (3,5)	4 (0,6)	0,217
OVSVE	382 (30,7)	99 (26,6)	201 (30,5)	82 (12,5)	0,013
SAM	374 (30,1)	121 (32,5)	192 (29,2)	61 (9,3)	0,457

BNP médio: * 405,0 ± 608 pg/mL, ^†^ 268,7 ± 299 pg/mL, ^‡^ 503,2 ± 689 pg/mL. Os valores são expressos por frequência e porcentagem. AE: átrio esquerdo; BNP: peptídeo natriurético tipo B; ECG: eletrocardiograma; FA: fibrilação atrial; FEVE: fração de ejeção do ventrículo esquerdo; OVSVE: obstrução da via de saída do ventrículo esquerdo; SAM: movimento anterior sistólico da valva mitral; SIV: septo interventricular.

**Tabela 4:** – Análises univariada e multivariada de mortalidade

	Univariada		Multivariada	
Variáveis	OR (IC 95%)	p valor	OR (IC 95%)	p valor
Sexo masculino	1,58 (1,18-2,10)	<0,001	1,20 (0,80-1,81)	0,375
Idade	1,04 (1,03-1,05)	<0,001	1,04 (1,03-1,06)	<0,001
FA/flutter atrial	0,58 (0,35-0,97)	0,038	2,14 (1,08-4,26)	0,029
FEVE	0,96 (0,94-0,97)	<0,001	0,97 (0,95-0,99)	0,005
AE	1,07 (1,05-1,09)	<0,001	1,05 (1,02-1,08)	<0,001

AE: átrio esquerdo; FA: fibrilação atrial; FEVE: fração de ejeção do ventrículo esquerdo; IC: intervalo de confiança; OR: odds ratio. Os modelos pertencem à regressão logística.

**Figura 3:**
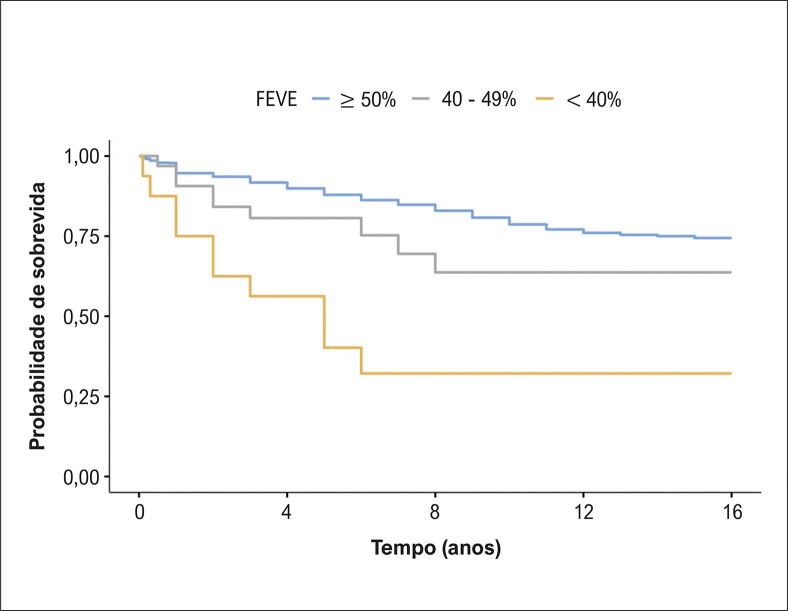
– Curvas de Kaplan-Meier para óbitos de acordo com FEVE. Teste de log-rank, p = 0,001. FEVE: fração de ejeção do ventrículo esquerdo.

Na avaliação baseada nos diferentes estratos de FEVE, o presente estudo revelou que no grupo com FEVE preservada (≥ 50%) foram incluídos 1196 pacientes, e a mortalidade ocorreu em 17,8%. No grupo de FEVE levemente reduzida (40% a 49%), foram incluídos 30 pacientes e a mortalidade foi de 30%. No grupo com FEVE reduzida (< 40%), foram incluídos 18 pacientes, com mortalidade de 55,5%. Houve diferença significativa na mortalidade (p = 0,001) de acordo com estratificação da FEVE, conforme demonstramos na Figura 3. Na Figura Central descrevemos os principais achados e a relação com prognóstico.

**Figura Central:**
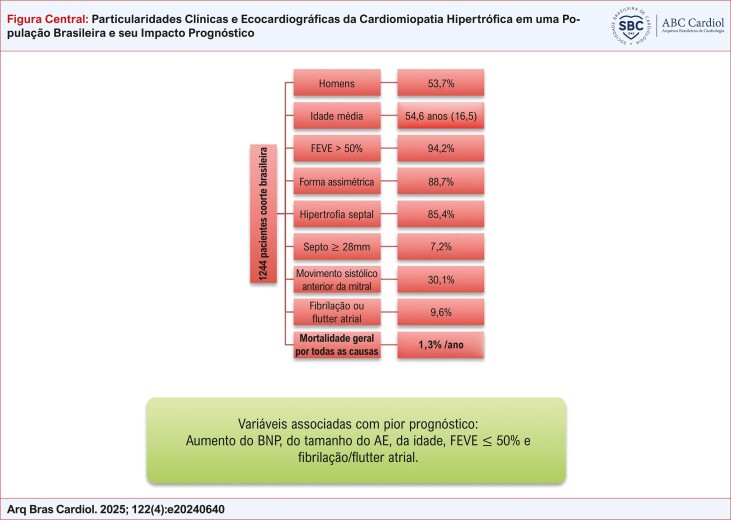
: Particularidades Clínicas e Ecocardiográficas da Cardiomiopatia Hipertrófica em uma População Brasileira e seu Impacto Prognóstico

AE: átrio esquerdo; BNP: peptídeo natriurético tipo B; FEVE: fração de ejeção do ventrículo esquerdo

## Discussão

Nosso estudo revelou que a maioria dos pacientes avaliados eram homens, com FEVE > 50%, com hipertrofia do tipo assimétrica e de predomínio septal. A taxa de mortalidade geral anual foi de 1,3%. O aumento do BNP, da idade, do tamanho do AE, a FEVE ≤ 50%, e a presença de fibrilação ou flutter atrial foram associados com pior prognóstico.

O paciente com CMH apresenta alterações estruturais e funcionais que podem ocasionar insuficiência cardíaca, dor torácica, arritmias, síncope e até morte súbita. Nesse contexto, o ETT é um exame fundamental para o diagnóstico, avaliação e acompanhamento desses pacientes. Além disso, permite identificar variáveis que podem estar relacionadas com o aumento da mortalidade.^2,8,19,20^

O sexo masculino (53,7%) apresentou um percentual discretamente maior do que o feminino, quando comparado a estudos prévios realizados em populações da América do Norte e da Europa, que evidenciaram uma população masculina podendo atingir de 62% a 71%. Provavelmente, por não termos muitos critérios de exclusão significativos, a frequência entre os sexos foi muito semelhante, refletindo nossa realidade no atendimento diário.^7,9-12^

A FEVE é frequentemente normal ou aumentada na CMH.^7^ Além disso, o tamanho do septo ≥ 28 mm, a presença de aneurisma apical e FEVE ≤ 50% são características que estão relacionados com risco de morte súbita.^8^ Em nossa população, 94% dos pacientes apresentaram FEVE superior a 50%. Esses achados estão em concordância com a literatura, que indica que menos de 10% dos pacientes apresentam FEVE ≤ 50%.^8^ Nosso estudo revelou que 7,2% dos pacientes apresentavam espessura septal ≥ 28 mm, grupo que merece atenção especial. Com relação ao aneurisma do VE, a literatura descreve que é um achado raro, entre 2% e 5% e pode estar relacionado a um maior risco de arritmias.^8^ Em nosso estudo, o aneurisma ventricular foi raríssimo, evidenciado em apenas 1 paciente. Provavelmente, foi subestimado pela limitação da avaliação pelo ecocardiograma não contrastado, principalmente nos casos de pequenos aneurismas. Além disso, a utilização da ressonância magnética melhorou a acurácia diagnóstica dessa alteração.^21-31^

Dados de literatura em populações mundiais revelam que cerca de 2/3 dos pacientes com CMH apresentam a forma obstrutiva. Neste estudo, apenas cerca de um terço da coorte avaliada apresentou a forma obstrutiva. Porém, por tratar-se de estudo de banco de dados e pelo fato do gradiente da via de saída do VE apresentar um componente dinâmico importante, a não realização ou realização de forma não eficaz da manobra de Valsalva durante a realização do ETT poderiam explicar estes achados.^2-8^

O BNP elevado aumenta o risco de eventos cardiovasculares em indivíduos com CMH.^8,13,14^ Neste contexto, Geske et al., em estudo com pacientes com CMH, demonstraram que o BNP foi um preditor independente de morbidade e mortalidade. Essas evidências são compatíveis com nossos resultados, que revelaram que o BNP > 200 pg/mL esteve relacionado à mortalidade.

A fibrilação ou flutter atrial podem ocorrer nos pacientes com CMH e estão associados ao agravamento do quadro funcional, a eventos tromboembólicos e ao aumento da mortalidade.^16-22^ O surgimento desta arritmia na CMH é multifatorial, porém está relacionada ao aumento do AE, achado comum nos pacientes com CMH. Nosso estudo revelou que 42,4% de todos os pacientes apresentavam um aumento do AE, sendo que nos pacientes mais idosos essa frequência é ainda maior, chegando a 50% nos pacientes com mais de 70 anos. Um estudo estadunidense publicado em 2014 identificou que a prevalência de fibrilação atrial em pacientes com CMH foi de 18%.^23^ Nosso trabalho identificou um número menor (9,6%) de pacientes com fibrilação ou flutter atrial. Uma das explicações para essa diferença foi a utilização do ECG da primeira avaliação clínica. Entretanto, sabemos que muitos pacientes podem evoluir com fibrilação atrial ao longo do acompanhamento.

Estudos prévios evidenciam que as mulheres com CMH são mais propensas a apresentar sintomas de insuficiência cardíaca, particularmente dispneia de esforço, fadiga, palpitações, dor no peito e classe funcional III a IV da New York Heart Association, em comparação aos homens. Além disso apresentam maior risco de eventos relacionados a doença, quando comparadas aos homens.^26,27^ Nosso estudo não avaliou a diferença nos sintomas ou desfecho clínico relacionados ao sexo. Avaliamos apenas os critérios do ecocardiograma, que revelou que as mulheres apresentavam tamanhos significativamente menores do septo, do diâmetro do AE, da parede posterior e dos diâmetros do VE.^25-28^Esses resultados que encontramos podem sugerir uma divergência com a literatura. Entretanto, necessitamos aprofundar melhor em estudos futuros, para verificar se essas alterações ecocardiográficas têm correlação com sintomas e desfechos.

Estudos revelam que 46% dos pacientes podem apresentar uma evolução benigna, com uma expectativa de vida normal e sem limitações. Os eventos adversos que encontramos em uma parcela dos pacientes incluem morte súbita, dor torácica, insuficiência cardíaca e fibrilação atrial.^8^ Intervenções cardiovasculares, como o implante de cardioversor desfibrilador implantável, reduziram as taxas de mortalidade cardiovascular para < 1,0%/ano.^32^ Nosso estudo apresentou mortalidade geral de 1,3%/ano. Essa diferença pode ser explicada, pois utilizamos para análise a mortalidade por todas as causas e não apenas a mortalidade cardiovascular. Além disso, por ser um centro de referência, os pacientes geralmente apresentam maior complexidade.^8,9^

Em um estudo, Chen et al.^33^ avaliaram 3605 pacientes chineses, em um período de acompanhamento médio de 4,6 anos e encontraram uma taxa de mortalidade por todas as causas de 6,3%. Em outro estudo chinês, Kwak et al.^34^ demonstraram que no grupo mais idoso, com idade média de 68 anos, a mortalidade por todas as causas em 5 anos foi de 12%. Nosso tempo médio de acompanhamento foi de 7,7 anos, com mortalidade de 18,5%. Além de serem populações distintas, uma das justificativas pela diferença provavelmente é devido ao acompanhamento mais longo. Estudo realizado na China, por Ma et al.,^35^ ao avaliar 2268 pacientes, revelou que a idade, a FEVE e o NT-proBNP foram preditores independentes de mortalidade por todas as causas. Nosso estudo também relacionou esses fatores como significativos para o prognóstico.

Os portadores de CMH apresentam maior morbimortalidade, principalmente quando encontramos fatores de risco associados. Entender as características da doença em nossa população permite uma compreensão mais precisa da enfermidade, com base nos dados nacionais. Novos estudos nacionais devem ser realizados para avaliar de forma prospectiva as características e evolução cardiovascular.

### Limitações do estudo

Trata-se de estudo retrospectivo baseado em dados de prontuário, o que limita a coleta de alguns dados clínicos e ecocardiográficos. A realização da investigação em um centro único, apesar de representar um hospital de referência, está sujeita ao viés de seleção dos participantes, que muito provavelmente reflete pacientes com o espectro clínico mais grave desta doença. As medidas das cavidades avaliadas pelo ecocardiograma não foram indexadas. A dosagem do BNP não estava disponível para análise em todos os casos. Outra limitação é que não tivemos acesso à causa da morte de cada paciente e por isso avaliamos a mortalidade por todas as causas.

## Conclusões

O estudo revelou que a maioria dos pacientes apresentava FEVE preservada, com hipertrofia assimétrica e de predomínio septal. A mortalidade geral, por todas as causas, foi de 1,3%/ano. O aumento do BNP, da idade, do tamanho do AE, a FEVE ≤ 50% e a presença de fibrilação ou flutter atrial foram associados com pior prognóstico.

## References

[1] Ommen SR, Ho CY, Asif IM, Balaji S, Burke MA, Day SM (2024). 2024 AHA/ACC/AMSSM/HRS/PACES/SCMR Guideline for the Management of Hypertrophic Cardiomyopathy: A Report of the American Heart Association/American College of Cardiology Joint Committee on Clinical Practice Guidelines. Circulation.

[2] Arbelo E, Protonotarios A, Gimeno JR, Arbustini E, Barriales-Villa R, Basso C (2023). 2023 ESC Guidelines for the Management of Cardiomyopathies. Eur Heart J.

[3] Kwon S, Kim HK, Kim B, Lee HJ, Han KD, Hwang IC (2022). Comparison of Mortality and Cause of Death between Adults with and without Hypertrophic Cardiomyopathy. Sci Rep.

[4] Lorenzini M, Anastasiou Z, O'Mahony C, Guttman OP, Gimeno JR, Monserrat L (2020). Mortality Among Referral Patients with Hypertrophic Cardiomyopathy vs the General European Population. JAMA Cardiol.

[5] Jacobsen MB, Petersen JK, Modin D, Butt JH, Thune JJ, Bundgaard H (2022). Long Term Mortality in Patients with Hypertrophic Cardiomyopathy - A Danish Nationwide Study. Am Heart J Plus.

[6] Wang Y, Gao W, Han X, Jiang J, Sandler B, Li X (2023). Cardiovascular Outcomes by Time-Varying New York Heart Association Class Among Patients with Obstructive Hypertrophic Cardiomyopathy: A Retrospective Cohort Study. J Med Econ.

[7] Lu DY, Pozios I, Haileselassie B, Ventoulis I, Liu H, Sorensen LL (2018). Clinical Outcomes in Patients with Nonobstructive, Labile, and Obstructive Hypertrophic Cardiomyopathy. J Am Heart Assoc.

[8] Fernandes F, Simões MV, Correia EB, Marcondes-Braga FG, Coelho-Filho OR, Mesquita CT (2024). Guidelines on the Diagnosis and Treatment of Hypertrophic Cardiomyopathy - 2024. Arq Bras Cardiol.

[9] Maron BJ, Rowin EJ, Udelson JE, Maron MS (2018). Clinical Spectrum and Management of Heart Failure in Hypertrophic Cardiomyopathy. JACC Heart Fail.

[10] Habib M, Adler A, Fardfini K, Hoss S, Hanneman K, Rowin EJ (2021). Progression of Myocardial Fibrosis in Hypertrophic Cardiomyopathy: A Cardiac Magnetic Resonance Study. JACC Cardiovasc Imaging.

[11] Melacini P, Basso C, Angelini A, Calore C, Bobbo F, Tokajuk B (2010). Clinicopathological Profiles of Progressive Heart Failure in Hypertrophic Cardiomyopathy. Eur Heart J.

[12] Neubauer S, Kolm P, Ho CY, Kwong RY, Desai MY, Dolman SF (2019). Distinct Subgroups in Hypertrophic Cardiomyopathy in the NHLBI HCM Registry. J Am Coll Cardiol.

[13] Matthia EL, Setteducato ML, Elzeneini M, Vernace N, Salerno M, Kramer CM (2022). Circulating Biomarkers in Hypertrophic Cardiomyopathy. J Am Heart Assoc.

[14] Minami Y, Haruki S, Kanbayashi K, Maeda R, Itani R, Hagiwara N (2018). B-Type Natriuretic Peptide and Risk of Sudden Death in Patients with Hypertrophic Cardiomyopathy. Heart Rhythm.

[15] Geske JB, McKie PM, Ommen SR, Sorajja P (2013). B-Type Natriuretic Peptide and Survival in Hypertrophic Cardiomyopathy. J Am Coll Cardiol.

[16] Rowin EJ, Link MS, Maron MS, Maron BJ (2023). Evolving Contemporary Management of Atrial Fibrillation in Hypertrophic Cardiomyopathy. Circulation.

[17] Mistrulli R, Ferrera A, Muthukkattil ML, Battistoni A, Gallo G, Barbato E (2024). Atrial Fibrillation in Patients with Hypertrophic Cardiomyopathy and Cardiac Amyloidosis: From Clinical Management to Catheter Ablation Indication. J Clin Med.

[18] Guttmann OP, Rahman MS, O'Mahony C, Anastasakis A, Elliott PM (2014). Atrial Fibrillation and Thromboembolism in Patients with Hypertrophic Cardiomyopathy: Systematic Review. Heart.

[19] Garg L, Gupta M, Sabzwari SRA, Agrawal S, Agarwal M, Nazir T (2019). Atrial Fibrillation in Hypertrophic Cardiomyopathy: Prevalence, Clinical Impact, and Management. Heart Fail Rev.

[20] Klopotowski M, Kwapiszewska A, Kukula K, Jamiolkowski J, Dabrowski M, Derejko P (2018). Clinical and Echocardiographic Parameters as Risk Factors for Atrial Fibrillation in Patients with Hypertrophic Cardiomyopathy. Clin Cardiol.

[21] Olivotto I, Cecchi F, Casey SA, Dolara A, Traverse JH, Maron BJ (2001). Impact of Atrial Fibrillation on the Clinical Course of Hypertrophic Cardiomyopathy. Circulation.

[22] Kubo T, Baba Y, Ochi Y, Hirota T, Yamasaki N, Kawai K (2021). Clinical Significance of New-Onset Atrial Fibrillation in Patients with Hypertrophic Cardiomyopathy. ESC Heart Fail.

[23] Siontis KC, Geske JB, Ong K, Nishimura RA, Ommen SR, Gersh BJ (2014). Atrial Fibrillation in Hypertrophic Cardiomyopathy: Prevalence, Clinical Correlations, and Mortality in a Large High-Risk Population. J Am Heart Assoc.

[24] Chumakova OS, Baklanova TN, Milovanova NV, Zateyshchikov DA (2023). Hypertrophic Cardiomyopathy in Underrepresented Populations: Clinical and Genetic Landscape Based on a Russian Single-Center Cohort Study. Genes.

[25] Butters A, Lakdawala NK, Ingles J (2021). Sex Differences in Hypertrophic Cardiomyopathy: Interaction with Genetics and Environment. Curr Heart Fail Rep.

[26] Zhao H, Tan Z, Liu M, Yu P, Ma J, Li X (2023). Is There a Sex Difference in the Prognosis of Hypertrophic Cardiomyopathy? A Systematic Review and Meta-Analysis. J Am Heart Assoc.

[27] Rowin EJ, Maron MS, Wells S, Patel PP, Koethe BC, Maron BJ (2019). Impact of Sex on Clinical Course and Survival in the Contemporary Treatment Era for Hypertrophic Cardiomyopathy. J Am Heart Assoc.

[28] Geske JB, Ong KC, Siontis KC, Hebl VB, Ackerman MJ, Hodge DO (2017). Women with Hypertrophic Cardiomyopathy Have Worse Survival. Eur Heart J.

[29] Maron MS, Rowin E, Spirito P, Maron BJ (2023). Differing Strategies for Sudden Death Prevention in Hypertrophic Cardiomyopathy. Heart.

[30] Arteaga E, Araújo AQ, Bernstein M, Ramires FJ, Ianni BM, Fernandes F (2009). Prognostic Value of the Collagen Volume Fraction in Hypertrophic Cardiomyopathy. Arq Bras Cardiol.

[31] Lee DZJ, Montazeri M, Bataiosu R, Hoss S, Adler A, Nguyen ET (2022). Clinical Characteristics and Prognostic Importance of Left Ventricular Apical Aneurysms in Hypertrophic Cardiomyopathy. JACC Cardiovasc Imaging.

[32] Maron BJ, Desai MY, Nishimura RA, Spirito P, Rakowski H, Towbin JA (2022). Management of Hypertrophic Cardiomyopathy: JACC State-of-the-Art Review. J Am Coll Cardiol.

[33] Chen QF, Zou J, Katsouras CS, You S, Zhou J, Ge HB (2024). Clinical Characteristics and Outcomes in Patients with Apical and Nonapical Hypertrophic Cardiomyopathy. J Am Heart Assoc.

[34] Kwak S, Kim J, Park CS, Lee HJ, Park JB, Lee SP (2024). Distinct Phenotypic Groups and Related Clinical Outcomes in Patients with Hypertrophic Cardiomyopathy. J Am Heart Assoc.

[35] Ma H, Zhou Y, He Y, Yu C, Liao Q, Xi H (2023). Prognosis for Patients with Apical Hypertrophic Cardiomyopathy: A Multicenter Cohort Study Based on Propensity Score Matching. Kardiol Pol.

