# Supplementary material for: Particularidades Clínicas e Ecocardiográficas da Cardiomiopatia Hipertrófica em uma População Brasileira e seu Impacto Prognóstico
Source: Arq Bras Cardiol. 2025 Apr 17;122(4):e20240640. [Article in Portuguese] doi: 10.36660/abc.20240640 (PMC12107843; doi:10.36660/abc.20240640)
Supplement: Translation

# Translation: Clinical and Echocardiographic Particularities of Hypertrophic Cardiomyopathy in a Brazilian Population and its Prognostic Impact

**DOI:** 10.36660/abc.20240640i

**Authors:** Georgina Del Cisne Jadán Luzuriaga, Edmundo Arteaga-Fernandez, Viviane Tiemi Hotta, Barbara Ianni, Luciano Nastari, Felix Ramires, Guilherme Wesley Peixoto da Fonseca, Charles Mady, Fábio Fernandes, Juliano Novaes Cardoso

**Affiliations:** 1 Brazil Instituto do Coração do Hospital das Clínicas da Faculdade de Medicina da Universidade de São Paulo, São Paulo, SP – Brazil; 2 Brazil Escola de Educação Física e Desporto da Universidade de São Paulo, São Paulo, SP – Brazil; 3 Brazil Universidade de São Paulo Faculdade de Medicina – Unidade Clínica de Miocardiopatias e Doenças da Aorta, São Paulo, SP – Brazil; 4 Brazil Faculdade Santa Marcelina – Cardiologia, São Paulo, SP – Brazil; 5 Brazil Hospital Santa Marcelina, São Paulo, SP – Brazil

**Keywords:** Hypertrophic Cardiomyopathy, Mortality, Echocardiography, Stroke Volume

## Abstract

**Background:**

Hypertrophic cardiomyopathy (HCM) presents echocardiographic abnormalities that are important for diagnosis and prognosis. Data are scarce in the Brazilian literature.

**Objective:**

To assess clinical and echocardiographic characteristics and disease progression in a Brazilian cohort of patients with HCM.

**Methods:**

This retrospective cohort included patients with HCM aged ≥ 18 years. Patients with moderate or severe aortic stenosis and those undergoing septal reduction were excluded. The significance level adopted in the statistical analysis was 5%.

**Results:**

The study included 1244 patients, between 2010 and 2022, with a mean follow-up time of 7.7 ± 4.5 years; 53.6% of patients were men. Mean age was 54.6 ± 16.5 years, and mean left ventricular ejection fraction (LVEF) was 65.8% ± 7.6. We observed LVEF ≤ 50% in 5.8% of patients, asymmetric form in 88.7%, and septal hypertrophy in 85.4%. We found systolic anterior motion of the mitral valve in 30.1% of patients, left ventricular outflow tract obstruction in 30.7%, and septum ≥ 28 mm in 7.2%. Only 1 patient had ventricular aneurysm. Atrial fibrillation/flutter occurred in 9.6% of patients. Overall mortality occurred in 232 patients (1.3%/year). Patients with B-type natriuretic peptide (BNP) > 200 pg/ml, left atrium ≥ 45 mm, and LVEF ≤ 50% had higher mortality (p < 0.001). Age and atrial fibrillation/flutter were also associated with mortality.

**Conclusions:**

The majority of patients had LVEF > 50%, asymmetric hypertrophy, and septal predominance. BNP, LA diameter, LVEF ≤ 50%, age, and atrial fibrillation/flutter were associated with worse prognosis.

## Introduction

Hypertrophic cardiomyopathy (HCM) is a hereditary disease, phenotypically characterized by myocardial hypertrophy that cannot be explained by systemic conditions, such as systemic arterial hypertension or metabolic or syndromic abnormalities.^1,2^ In adults, diagnosis is defined by diastolic myocardial thickness ≥ 15 mm in any location of the left ventricle (LV), or ≥ 13 mm in individuals with a family history of first-degree relatives with a confirmed diagnosis of HCM.^2-8^

Transthoracic echocardiography (TTE) is an accessible and essential exam for initial diagnosis, risk stratification, and clinical follow-up in HCM.^8^ The method makes morphological and functional assessment of the heart possible, in addition to identifying, quantifying, and localizing ventricular hypertrophy. It also allows for assessment of the presence of left ventricular outflow tract obstruction (LVOTO), systolic anterior motion of the mitral valve (SAM), apical aneurysm, or intracavitary masses.^2,8^

HCM has two clinical forms, obstructive and non-obstructive, according to the presence of LVOTO, defined by gradient measurement ≥ 30 mmHg at rest or ≥ 50 mmHg after provocative maneuvers, such as the Valsalva maneuver.^8^ Multiple characteristics assessed on TTE are related to prognosis, for example, left atrial (LA) dimensions, maximum septal thickness, left ventricular ejection fraction (LVEF), and the presence of LV apical aneurysm. The disease can, in some cases, cause symptoms such as dyspnea, chest pain, syncope, and palpitations. In patients considered high risk for sudden death, HCM may be associated with higher mortality.^2,8,9-18^

Our study aimed to add knowledge related to patients with HCM in Brazil, allowing us to better understand their peculiarities. Due to the importance of TTE in the initial assessment, risk stratification, and follow-up of patients with HCM, this study aimed to analyze the clinical and echocardiographic characteristics and progression in a Brazilian cohort of patients with HCM. Even though it is a genetic disease with a hereditary pattern, knowledge of particularities related to demographic, socioeconomic, and environmental variations in the clinical expression of HCM is fundamental.]

## Material and methods

This study evaluated a retrospective cohort of patients diagnosed with HCM followed at a tertiary hospital in the city of São Paulo, Brazil, during the period from 2010 to 2022, using electronic medical record data. The study excluded patients under 18 years of age; patients with aortic stenosis; patients undergoing septal reduction therapy, heart valve surgery, or heart transplantation; and those with other cardiomyopathies.

We analyzed demographic, clinical, and laboratory data, as well as rhythm on resting electrocardiogram, TTE, 24-hour Holter, and all-cause mortality.

### Echocardiography and LVEF measurement

The measurements of cardiac cavities and dimensions, including LA diameter, LV wall diameters, and myocardial thickness, were performed in 2-dimensional mode in the longitudinal parasternal view. Regarding measurements of the ventricular septum, we excluded right ventricular structures such as trabeculations, moderator band, and supraventricular crest, as well as papillary muscles. HCM was considered to be asymmetric when the difference between 1 or more segments with LV myocardial hypertrophy was > 2 mm assessed on TTE. LVOTO was defined as the presence of LV outflow tract gradient ≥ 30 mmHg at rest and ≥ 50 mmHg after Valsalva maneuver.^1,7^ Exams were performed by experienced physicians at a cardiology referral hospital using equipment from different brands over a 12-year follow-up period. The examinations were performed using equipment from GE Healthcare Systems (Vivid I, Vivid E9, Vivid E95, iQ) and Phillips Healthcare (ie 33, Epic, CX, and CVX).

### Statistical analysis

For statistical processing of the data, SPSS V26 (2019), Minitab 21.2 (2022), and Office Excel 2010 were used. We used unpaired Student’s t test for continuous variables and the chi-square test for categorical variables. The Shapiro-Wilk test was used to test the normality of continuous variables. Survival curves were analyzed using the Kaplan-Meier method, and differences in death rates over time were assessed using the log-rank test. Logistic regression models were constructed to assess factors associated with mortality. Odds ratios (OR) and 95% confidence intervals (CI) were recorded. Significant associations in the univariate analysis were included in the multivariate analysis model. Continuous variables were presented as mean ± standard deviation, and categorical variables were shown as frequency and percentage. The significance level adopted in the statistical analysis was 5%. This study received approval from the institution’s ethics committee.

## Results

The study included 1244 patients in the period from 2010 to 2022, with a mean follow-up time of 7.7 ± 4.5 years. The mean age was 54.6 ± 16.5 years, and 53.6% were male. Atrial fibrillation or flutter was present in 9.6% of patients. Table 1 describes the clinical and laboratory characteristics.

**Table 1:** – General clinical and laboratory characteristics of the total study population

	All patients n = 1244
**Age, years (SD)**	54.6 (16.5)
**Sex**	
Male (%)	667 (53.6%)
Female (%)	577 (46.4%)
**Race**	
White (%)	1.102 (88.6)
Black (%)	67 (5.4)
Mixed (%)	61 (4.9)
Asian (%)	14 (1.1)
**Vital signs**	
HR (bpm)	64.9 ± 19.3
SBP (mmHg)	114.8 ± 33.7
DBP (mmHg)	71.5 ± 20.7
**Laboratory exams**	
BNP, pg/mL*	347.7 ± 492.7
Hemoglobin, g/dL	14.3 ± 1.7
Glucose, mg/dL	104.2 ± 29.3
LDL, mg/dL	116.2 ± 37.9
Urea, mg/dL	36.9 ± 13.3
Creatinine, mg/L	1.0 ± 0.3
Potassium, mEq/L	4.4 ± 0.4
Sodium, mEq/L	139.9 ± 2.8
**ECG ^**†**^**	
Sinus rhythm (%)	712 (85.6)
AF/flutter (%)	80 (9.6)
Others (%)	40 (4.8)

* 853 patients. ^†^ 832 patients. Values are expressed as mean ± SD or frequency and percentage. AF: atrial fibrillation; BNP: B-type natriuretic peptide; DBP: diastolic blood pressure; ECG: electrocardiogram; HR: heart rate; LDL: low-density lipoprotein; SBP: systolic blood pressure; SD: standard deviation.

TTE assessment revealed mean LA diameter of 43.7 ± 7.3 mm and mean LVEF of 65.8% ± 7.6, with no statistical difference between sexes (p = 0.498). LVEF > 50% was observed in 1172 patients (94.2%), whereas LVEF equal to or less than 50% was observed in only 72 patients (5.8%). The asymmetric form occurred in 1104 patients (88.7%); 1062 patients (85.4%) had predominantly septal hypertrophy, and 34 patients (2.7%) had predominantly apical hypertrophy. Ventricular septal thickness ≥ 28 mm was observed in 89 patients (7.2%), and apical ventricular aneurysm was identified in only 1 patient. SAM was found in 374 patients (30.1%), and LVOTO was observed in 382 patients (30.7%).

There was a difference between sexes when comparing echocardiographic assessment of the following parameters: LA diameter, interventricular septum, posterior wall, and left ventricular volume (Table 2). In the evaluation of age groups, patients aged > 70 years had a higher frequency of BNP above 200 pg/mL, atrial fibrillation or flutter, and LA size ≥ 45 mm (Table 3).

**Table 2:** – Echocardiographic characteristics of the total study population and by sex

	All patients (n = 1244)	Men (n = 667)	Women (n = 577)	p value
**LVEF**	65.8 ± 7.6	65.7 ± 7.7	66.1 ± 6.3	0.498
**LA (mm)**	43.7 ± 7.3	44.3 ± 7.9	43.0 ± 7.3	0.003
**IVS (mm)**	18.9 ± 5.7	19.3 ± 6.0	18.8 ± 5.6	0.002
**LVPW (mm)**	11.4 ± 2.7	11.6 ± 2.8	11.0 ± 2.8	0.012
**LVDD (mm)**	45.1 ± 6.0	46.3 ± 6.3	43.9 ± 5.5	<0.001
**LVSD (mm)**	28.7 ± 5.3	29.4 ± 5.7	27.8 ± 4.5	<0.001
**LVDV (ml)**	96.3 ± 40.0	103.3 ± 48.4	89.2 ± 26.4	<0.001
**LVSV, (ml)**	33.5 ± 17.8	35.8 ± 19.7	30.5 ± 12.4	<0.001
**LVMI (g/m^2^)**	161.6 ± 64.1	164.4 ± 68.8	160.9 ± 60.6	0.100
**Thickness index (mm)**	0.7 ± 0.2	0.7 ± 0.2	0.7 ± 0.2	0.109
**According to symmetry**				
Asymmetric (%)	1104 (88.7)	593 (88.9)	511 (88.6)	0.848
Symmetric (%)	140 (11.3)	74 (11.1)	66 (11.4)	
SAM (%)	374 (30.1)	198 (15.9)	176 (14.1)	0.754
**LVOTO**				
Present	382	174	208	<0.001
Absent	862	493	369	

Values are expressed as mean ± SD or frequency and percentage. IVS: interventricular septum; LA: left atrium; LVDD: left ventricular diastolic diameter; LVDV: left ventricular diastolic volume; LVEF: left ventricular ejection fraction; LVMI: left ventricular mass index; LVOTO: left ventricular outflow tract obstruction; LVPW: left ventricular posterior wall; LVSD: left ventricular systolic diameter; LVSV: left ventricular systolic volume; SAM: systolic anterior motion of the mitral valve.

**Table 3:** – Characteristics by age group

	All patients	18 to 45 years old	46 to 70 years old	≥ 71 years	p value
**BNP**	n=853 (%)	n=247*(%)	n=464†(%)	n=143^‡^(%)	
≤ 200 pg/mL	422 (49.5)	122 (49.4)	253 (54.6)	47 (32.9)	<0.001
201 to 500 pg/mL	271 (31.8)	69 (27.9)	146 (31.4)	57 (39.9)	
≥ 501 pg/mL	160 (18.8)	56 (22.7)	65 (14.0)	39 (27.3)	
**ECG rhythm**	n=832(%)	n=228 (%)	n=455 (%)	n=149 (%)	
Sinus	713 (85.7)	218 (95.6)	378 (83.1)	117 (78.5)	<0.001
AF/flutter	80 (9.6)	3 (1.3)	55 (12.1)	22 (14.8)	
Others	39 (4.7)	7 (3.1)	22 (4.8)	10 (6.7)	
**Echocardiogram**	n=1244(%)	n=372 (%)	n=658 (%)	n=214 (%)	
LA ≥ 45 mm	527 (42.4)	113 (30.4)	307 (46.7)	107 (50.0)	<0.001
IVS ≥ 28 mm	89 (7.2)	54 (14.5)	32 (4.9)	3 (1.4)	<0.001
LVEF < 50%	44 (3.5)	17 (4.6)	23 (3.5)	4 (0.6)	0.217
LVOTO	382 (30.7)	99 (26.6)	201 (30.5)	82 (12.5)	0.013
SAM	374 (30.1)	121 (32.5)	192 (29.2)	61 (9.3)	0.457

Mean BNP: * 405.0 ± 608 pg/mL, ^†^ 268.7 ± 299 pg/mL, ^‡^ 503.2 ± 689 pg/mL. Values are expressed as frequency and percentage. AF: atrial fibrillation; BNP: B-type natriuretic peptide; ECG: electrocardiogram; IVS: interventricular septum; LA: left atrium; LVEF: left ventricular ejection fraction; LVOTO: left ventricular outflow tract obstruction; SAM: systolic anterior motion of the mitral valve.

During follow-up, with a mean period of 7.7 years (minimum of 1 year and maximum of 16 years), the mortality rate was 18.6% (mean age of 62.0 ± 17.0 years, 53.9% female). The annual mortality rate was 1.3% among the patients studied. The survival curve revealed higher mortality for patients with BNP > 200 pg/mL (Figure 1), LA diameter ≥ 45 mm (Figure 2), and LVEF ≤ 50%, with log-rank test showing p < 0.001. Univariate analysis revealed that male sex was associated with risk of death (OR 1.58, 95% CI: 1.18 to 2.10, p < 0.001). We also performed multivariate analysis, which showed that the following variables were related to higher mortality: advanced age, increased LA diameter, reduced LVEF, and presence of atrial fibrillation or flutter (Table 4).

**Figure 1:** – Hypertrophic cardiomyopathy according to BNP values. Log-rank test, p < 0.001. BNP: B-type natriuretic peptide.

**Figure 2:** – Hypertrophic cardiomyopathy and left atrium ≥ 45mm. Log-rank test, p < 0.001.

**Table 4:** – Univariate and multivariate analyses of mortality

	Univariate		Multivariate	
Variables	OR (95% CI)	p value	OR (95% CI)	p value
Male sex	1.58 (1.18-2.10)	<0.001	1.20 (0.80-1.81)	0.375
Age	1.04 (1.03-1.05)	<0.001	1.04 (1.03-1.06)	<0.001
AF/flutter	0.58 (0.35-0.97)	0.038	2.14 (1.08-4.26)	0.029
LVEF	0.96 (0.94-0.97)	<0.001	0.97 (0.95-0.99)	0.005
LA	1.07 (1.05-1.09)	<0.001	1.05 (1.02-1.08)	<0.001

AF: atrial fibrillation; CI: confidence interval; LA: left atrium; LVEF: left ventricular ejection fraction; OR: odds ratio. The models belong to logistic regression.

In the assessment based on different LVEF strata, the group with preserved LVEF (≥ 50%) included 1196 patients, and mortality occurred in 17.8%. The group with slightly reduced LVEF (40% to 49%) included 30 patients, and mortality was 30%. The group with reduced LVEF (< 40%) included 18 patients, with mortality of 55.5%. There was a significant difference in mortality (p = 0.001) according to LVEF stratification, as shown in Figure 3. The Central Illustration describes the main findings and their relationship with prognosis.

**Figure 3:** – Kaplan-Meier curves for mortality according to LVEF. Log-rank test, p = 0.001. LVEF: left ventricular ejection fraction.

**Central Illustration:** : Clinical and Echocardiographic Particularities of Hypertrophic Cardiomyopathy in a Brazilian Population and its Prognostic Impact

BNP: B-type natriuretic peptide; LA: left atrial; LVEF: left ventricular ejection fraction.

## Discussion

In our study, the majority of patients evaluated were men, with LVEF > 50%, asymmetric hypertrophy, and septal predominance. The overall annual mortality rate was 1.3%. Increased BNP, advanced age, LA enlargement, LVEF ≤ 50%, and the presence of atrial fibrillation or flutter were associated with a worse prognosis.

Patients with HCM present structural and functional alterations that can lead to heart failure, chest pain, arrhythmias, syncope, and even sudden death. In this context, TTE is a fundamental exam for the diagnosis, assessment, and follow-up of these patients. Moreover, it allows the identification of variables that may be related to increased mortality.^2,8,19,20^

The male sex (53.7%) showed a slightly higher percentage than the female sex, when compared to previous studies conducted in populations from North America and Europe, which showed that the male population could reach 62% to 71%. Probably, because we did not have many significant exclusion criteria, the frequency between the sexes was very similar, reflecting our reality in daily care.^7,9-12^

In HCM, LVEF is often normal or increased.^7^ Furthermore, septal size ≥ 28 mm, the presence of apical aneurysm, and LVEF ≤ 50% are characteristics that are related to the risk of sudden death.^8^ In our study population, 94% of patients had LVEF greater than 50%. These findings are in agreement with the literature, which indicates that less than 10% of patients have LVEF ≤ 50%.^8^ Our study revealed that 7.2% of patients had septal thickness ≥ 28 mm, and this group deserves special attention. With respect to left ventricular aneurysm, the literature describes it as a rare finding, between 2% and 5%, and it may be related to a higher risk of arrhythmias.^8^ In our study, ventricular aneurysm was very rare, found in only 1 patient. It was probably underestimated due to the limited assessment by non-contrast echocardiography, especially in cases of small aneurysms. Moreover, the use of magnetic resonance imaging has been shown to improve the diagnostic accuracy for this alteration.^21-31^

Data from the literature on global populations reveal that approximately 2/3 of patients with HCM have the obstructive form. In our study, approximately 1/3 of the cohort evaluated had the obstructive form. However, given that this was a database study and that LV outflow tract gradient is an important dynamic component, failure to perform the Valsalva maneuver or ineffective performance of the maneuver during TTE could explain these findings.^2-8^

High BNP increases the risk of cardiovascular events in patients with HCM.^8,13,14^ In this context, Geske et al., in a study of patients with HCM, demonstrated that BNP was an independent predictor of morbidity and mortality. This evidence is consistent with our results, which revealed that BNP > 200 pg/mL was related to mortality.

Atrial fibrillation or flutter may occur in patients with HCM and are associated with worsening of functional status, thromboembolic events, and increased mortality.^16-22^ The onset of this arrhythmia in HCM is multifactorial, but it is related to LA enlargement, which a common finding in patients with HCM. Our study revealed that 42.4% of all patients had LA enlargement, and this frequency was even higher in older patients, reaching 50% in patients over 70 years of age. A study from the United States published in 2014 found that the prevalence of atrial fibrillation in patients with HCM was 18%.^23^ Our study identified a smaller number (9.6%) of patients with atrial fibrillation or flutter. One of the explanations for this difference was the use of electrocardiogram in the first clinical assessment. Nonetheless, we are aware that many patients may develop atrial fibrillation during follow-up.

Previous studies have shown that women with HCM are more likely to have symptoms of heart failure, particularly dyspnea on exertion, fatigue, palpitations, chest pain, and New York Heart Association functional class III to IV, when compared to men. Moreover, they have a higher risk of disease-related events when compared to men.^26,27^ Our study did not evaluate difference in symptoms or clinical outcomes related to sex. We only evaluated echocardiogram criteria, which revealed that women had significantly smaller septum, LA diameter, posterior wall, and LV diameters.^25-28^Our findings may suggest a divergence with the literature. However, more in-depth investigation is needed in future studies in order to verify whether these echocardiographic changes are correlated with symptoms and outcomes.

Studies have shown show that 46% of patients may have a benign course, with normal life expectancy and no limitations. The adverse events that are found in a portion of patients include sudden death, chest pain, heart failure, and atrial fibrillation.^8^ Cardiovascular interventions, such as implantable cardioverter-defibrillator implantation, have reduced cardiovascular mortality rates to less than 1.0%/year.^32^ Our study showed an overall mortality rate of 1.3%/year. This difference can be explained by the fact that we used all-cause mortality for analysis, rather than only cardiovascular mortality. Moreover, as the study was conducted at a referral center, patients generally present greater complexity.^8,9^

In one study, Chen et al.^33^ evaluated 3605 Chinese patients over a mean follow-up period of 4.6 years, and they found an all-cause mortality rate of 6.3%. In another study from China, Kwak et al.^34^ demonstrated that, in the oldest group, with a mean age of 68 years, all-cause mortality at 5 years was 12%. Our mean follow-up time was 7.7 years, with mortality of 18.5%. In addition to the fact that they are distinct populations, one of the justifications for this difference is likely due to our longer follow-up period. A study conducted in China by Ma et al.,^35^ assessing 2268 patients, revealed that age, LVEF, and NT-proBNP were independent predictors of all-cause mortality. Our study also reported that these factors were significant for prognosis.

Patients with HCM have higher morbidity and mortality, especially when we find associated risk factors. Understanding the characteristics of the disease in our population allows for more accurate comprehension, based on national data. New national studies should be conducted to prospectively evaluate cardiovascular characteristics and progression.

### Study limitations

This was a retrospective study based on medical records, which limits the collection of some clinical and echocardiographic data. Due to the fact that the investigation was conducted at a single center, even though it is a referral hospital, it is subject to participant selection bias, which most likely reflects patients on the most severe clinical spectrum of this disease. The cavity measurements assessed by echocardiography were not indexed. BNP levels were not available for analysis in all cases. Another limitation is that we did not have access to the cause of death for each patient and, therefore, assessed all-cause mortality.

## Conclusions

This study revealed that the majority of patients had preserved LVEF, with asymmetric hypertrophy and septal predominance. Overall all-cause mortality was 1.3%/year. Increased BNP, advanced age, LA enlargement, LVEF ≤ 50%, and the presence of atrial fibrillation or flutter were associated with worse prognosis.
